# Unstable EBV latency drives inflammation in multiple sclerosis patient derived spontaneous B cells

**DOI:** 10.21203/rs.3.rs-2398872/v1

**Published:** 2023-02-01

**Authors:** Samantha Soldan, Chenhe Su, Maria Chiara Monaco, Natalie Brown, Annaliese Clauze, Frances Andrada, Andries Feder, Paul Planet, Andrew Kossenkov, Daniel Schäffer, Joan Ohayon, Noam Auslander, Steve Jacobson, Paul Lieberman

**Affiliations:** Wistar Institute; Wistar Institute; National Institutes of Health - National Institute of Neurological Disorders and Stroke; Wistar Institute; NIH; NIH; Childrens Hospital of Philadelphia; Children's Hospital of Philadelphia; Wistar Institute; Computational Biology Department, Carnegie Mellon University; NIH; Wistar Institute; NINDS/NIH; Wistar Institute

**Keywords:** Multiple Sclerosis, Epstein-Barr Virus, latency, lytic activation, lymphoblastoid cell lines (LCLs)

## Abstract

Epidemiological studies have demonstrated that Epstein-Barr virus (EBV) is a known etiologic risk factor, and perhaps prerequisite, for the development of MS. EBV establishes life-long latent infection in a subpopulation of memory B cells. Although the role of memory B cells in the pathobiology of MS is well established, studies characterizing EBV-associated mechanisms of B cell inflammation and disease pathogenesis in EBV (+) B cells from MS patients are limited. Accordingly, we analyzed spontaneous lymphoblastoid cell lines (SLCLs) from multiple sclerosis patients and healthy controls to study host-virus interactions in B cells, in the context of an individual’s endogenous EBV. We identify differences in EBV gene expression and regulation of both viral and cellular genes in SLCLs. Our data suggest that EBV latency is dysregulated in MS SLCLs with increased lytic gene expression observed in MS patient B cells, especially those generated from samples obtained during “active” disease. Moreover, we show increased inflammatory gene expression and cytokine production in MS patient SLCLs and demonstrate that tenofovir alafenamide, an antiviral that targets EBV replication, decreases EBV viral loads, EBV lytic gene expression, and EBV-mediated inflammation in both SLCLs and in a mixed lymphocyte assay. Collectively, these data suggest that dysregulation of EBV latency in MS drives a pro-inflammatory, pathogenic phenotype in memory B cells and that this response can be attenuated by suppressing EBV lytic activation. This study provides further support for the development of antiviral agents that target EBV-infection for use in MS.

## Introduction

Epstein-Barr virus (EBV) is a ubiquitous human g-herpesvirus that establishes a stable, long-term latent infection in memory B-cells ^1-5^. Although most EBV infections are benign, genetic susceptibilities, immune-deficiencies, environmental exposures, and life-history events can enable various pathogenic outcomes, including diverse malignancies and immune disorders. EBV was the first human tumor virus described and has a well-established pathogenic role in cancers of B cell, NK/T cell, and epithelial origin including, Burkitt’s and Hodgkin’s lymphomas; NK/T cell lymphomas; primary CNS lymphomas ; nasopharyngeal carcinoma; and lymphoepithelial gastric carcinomas ^6-8^. In addition, EBV is associated with several autoimmune disorders, with the strongest risk correlation linked to multiple sclerosis (MS) ^9,10^

MS is a common and heterogeneous demyelinating disorder with a complex etiology that develops as a consequence of the interplay between the immune system and the environment in genetically susceptible individuals ^11,12^. Studies linking EBV to the etiology of MS began nearly forty years ago ^13^. Over the years, a preponderance of epidemiologic, immunologic, and virologic evidence has strengthened this association ^10,14^. There are several known abnormalities in the immune response to EBV in MS, including increased titers of EBV-specific antibodies, deficient cytotoxic T cell (CTL) control of EBV infection, and evidence of molecular mimicry between EBV-encoded proteins and CNS antigens ^10,15-19^. The age of acquiring EBV affects an individual’s risk for developing MS. When acquired early in life, EBV primary infection is typically asymptomatic or occurs with few noticeable symptoms ^20^. In contrast, primary infection in adolescence or early adulthood can result in infectious mononucleosis (IM) ^21^. IM is typically self-limiting and symptoms resolve in approximately two weeks as the immune system controls but does not eliminate EBV, allowing the virus to establish long-term latent infection as episomes in memory B cells ^22^. Throughout an individual’s lifetime, continuous T cell surveillance tightly controls EBV lytic reactivation, while latently infected B cells adeptly evade the cellular immune response ^23^. Sporadic EBV reactivation occurs in all healthy carriers but may be elevated prior to disease onset. MS risk is significantly correlated with later life EBV infection and severity of IM. However, the precise mechanisms by which EBV may act as a trigger and potentially, a driver of disease pathogenesis in MS are poorly defined ^9^.

In the last few decades, while evidence for a role of EBV as an environmental co-factor for MS was mounting, the understanding of the immunopathology of MS shifted considerably. Early studies suggested that MS was predominately a T-cell-mediated disease ^24^. However, more recent use of B cell depletion therapies has demonstrated that B cells are a key component of disease pathogenesis. Memory B cells and plasma blasts are predominantly detected in the CSF of MS patients and are rarely detected or undetectable in the CSF of normal donors^25^ Treatments that broadly target B cells, including anti-CD20 (ocrelizumab and ofatumumab), anti-CD52, and cladribine, are clinically effective in MS, while those that target naïve and plasma B cells (e.g., Atacicept) or boost memory B cells (e.g. infliximab) are deleterious ^26,27^. Furthermore, studies have identified an inflammatory B cell subpopulation secreting elevated levels of GM-CSF and IL-6 in MS patients ^27,28^. Moreover, epigenetic modification of cellular genes associated with inflammation in MS, including osteopontin and CXCR4 correlate with increase neuroinvasion of EBV (+) B cells^29^. These studies suggest that there is an inflammatory memory B cell population that specifically contributes to the pathobiology of MS, potentially the same B cell population that serves as the primary reservoir of latent EBV infection.

Although inflammatory B cells play a key role in disease pathogenesis and are the source of latent EBV infection, little is known about how host-virus interactions may differ in EBV infected B cells of MS patients compared to healthy individuals. Lymphoblastoid cell lines (LCLs) are immortalized human B cell lines obtained by infecting peripheral blood mononuclear cells (PBMCs) or purified B cells in vitro with an exogenous, laboratory strain of EBV (typically B95.8). While LCLs are commonly used to study latent EBV, they do not represent the virus endogenous to the individual from whom these B cell lines are derived. Previous studies have described the generation of spontaneous lymphoblastoid cell lines/SLCLs from MS patients, IM, and healthy controls by extended culture of PBMCs in the presence of cyclosporin A or anti-CD3 antibodies to eliminate T cells ^30,31^. We have generated a small cohort of SLCLs from healthy controls and MS patients derived from PBMC obtained during both “active” and “stable” disease, as defined by MRI. In this study, we analyze these SLCLs by whole genome sequence, RNA-seq transcriptomics, biochemical analysis of EBNA1 DNA binding activity, cytokine release, and T-cell immune activation. RNA-seq transcriptomics identified differentially regulated viral and cellular genes that may function as novel regulators of EBV latency. Our data suggest that MS patients fail to efficiently control EBV latency and that aberrant lytic activation drives B cell inflammatory phenotypes. Furthermore, treatment with tenofovir alafenamide (TAF), a nucleoside analogue that targets EBV active replication ^32^, decreased EBV lytic gene expression and attenuated inflammatory markers in MS patient SLCLs. Our study provides a new experimental model to understand the molecular basis for aberrant B-cell control of EBV latency, and further supports a direct role for EBV infected memory B cells in the pathobiology of MS.

## Results

### Generation, growth characteristics, and EBV viral loads of spontaneous lymphoblastoid cell lines (SLCLS) from MS patients and healthy controls

**Figure 1:**
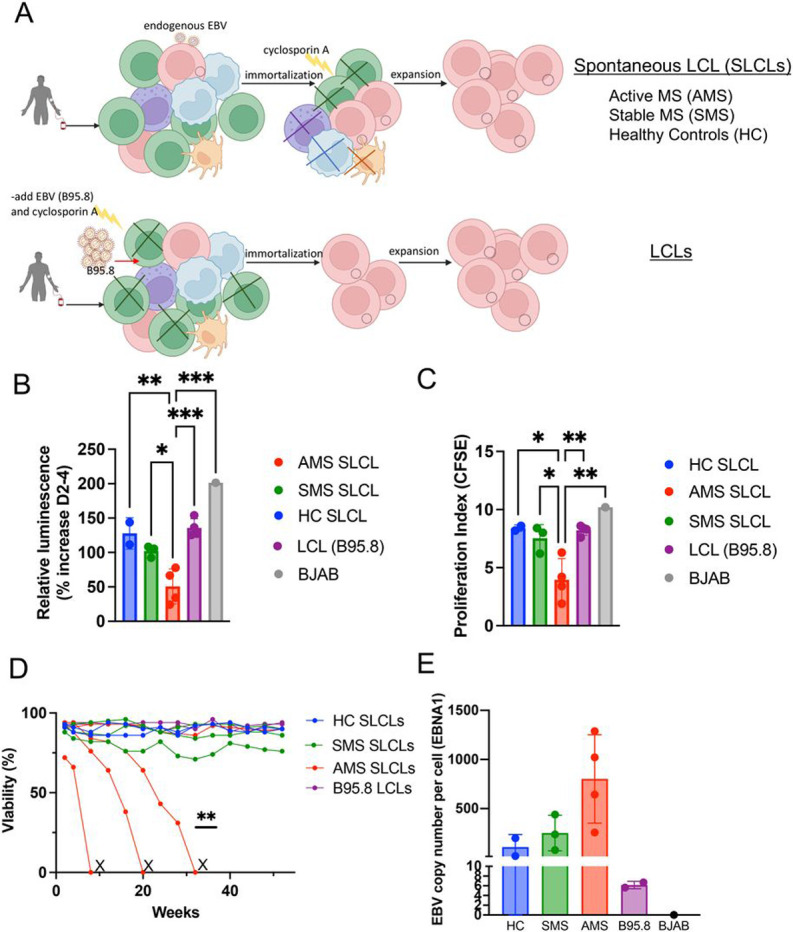
Generation and growth of SLCLs from the PBMC of MS patients and healthy controls (HC). (**A**) SLCLs were generated from PBMCs isolated from MS patients and healthy controls by extended culture of PBMCs without addition of exogenous, lab strain EBV. Three weeks after *ex vivo* culture, cyclosporin A was added to eliminate residual T -cells. (**B**) cell viability in SLCLs, LCLs (B95.8), and EBV (−) BJAB cells measured by Cell Titer-Glo^®^ Cell luminescent Cell Viability Assay (*p<0.05, **p<0.01, ***p<0.001; one-way ANOVA followed by Tukey’s multiple comparison test). (C) Proliferation index in SLCLs, LCLs (B95.8), and EBV (−) BJAB cells measured by CFSE (*p<0.05, **p<0.001; one-way ANOVA followed by Tukey’s multiple comparison test). (**D**) Viability of long-term culture of AMS SLCLs compared to HC and SMS SLCLs, LCLs (B95.8), and EBV (−) BJAB cells (**P=0.0023, Log-rank Mantel-Cox test).

In a related study, we describe the generation of SLCLs from healthy controls or MS patients with either active or stable disease, as defined by MRI. We enrolled 29 individuals: 14 healthy controls and 15 MS patients (Fig. 1A, Table 1). From these individuals, we were able to propagate SLCLs from 2 (14%) of healthy controls (HCs) and 46% of MS patients: 43% of PBMCs isolated from MS patients during stable disease (SMS) and 50% of PBMCs isolated from MS patients during active disease (AMS). The increased yield of SLCLs from patients with MS compared to healthy controls is consistent with previous reports ^33 34^

We first evaluated SLCLs for their growth characteristics compared to laboratory strain-derived LCLs (B95.8) and the EBV (−) cell line BJAB (Fig. 1B-D). The Cell Titer-Glo^®^ Cell luminescent Cell Viability Assay (Promega) was used to determine the relative number of metabolically active cells by quantifying the amount of ATP present on days 2 and 4 in culture. The cell viability assay revealed decreased metabolic activity, reflecting a proportionate decrease in cell number, in SLCLs derived from AMS patients compared to SMS and HC SLCLs, LCLs (B95.8), and BJABs (Fig. 1B). To further define the growth characteristics of SLCs, we performed a CFSE cell proliferation assay to determine the proliferation index of each cell line. Again, cell growth was markedly slower in SLCLs derived from AMS (Fig. 1C). Moreover, when we maintained extended cultures of SLCLs, LCLs and BJABs (up to one year), we noted that SLCLs derived from AMS patients were more difficult to maintain in culture than SLCLs from SMS patients or HC and three out of four of the AMS lines could not be sustained for one year (Fig. 1D). Notably, EBV DNA copy number per cell, as determined by qPCR, trended higher in SLCLs derived from patients with active MS than in SLCLs derived from healthy controls or patients with stable disease (Fig. 1E).

In addition, flow cytometry revealed that SLCLs were slightly smaller than exogenously (B95.8) transformed LCLs as determined by forward scatter (FSC) (**Supplemental Fig. 1A and B**) and cell surface staining confirmed that all SCLs were CD19+, CD20 + and complement receptor 2 (CR2; CD21) + B cells (Supplemental Fig. 1C and D). The EBV (−) cell line BJAB does not express the EBV receptor CD21. Of interest, CD21 was present but downregulated in cells from AMS SLCLs (Supplemental Fig. 1C). Some viruses have been observed to downregulate their receptors during infection. For example, membrane cofactor protein (MCP; CD46) is downregulated by viruses that use this molecule as a receptor, including human herpesvirus type 6 and measles virus ^35,36^ and EBV has been shown to downregulate CD21 in immature thymocytes ^37^. No other notable differences in cell surface marker expression (CD19, CD20, CD11c, CD80, and CD86) were identified (Supplemental Fig. 1E).

### Increased lytic gene expression in SLCLs derived from AMS patients

**Figure 2:**
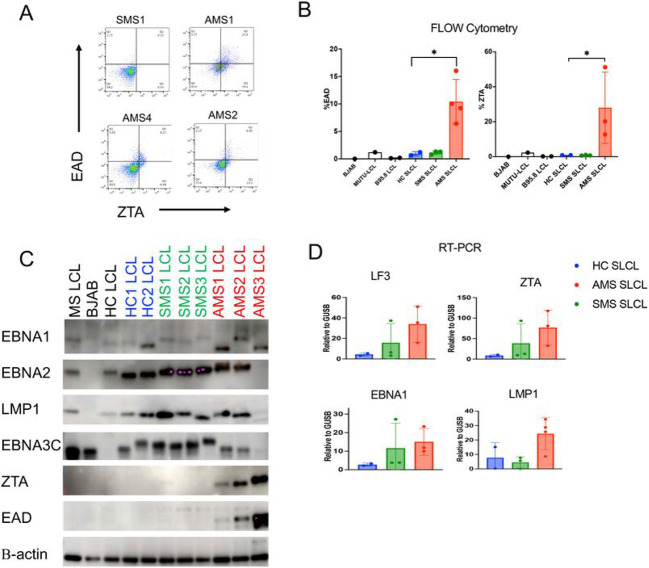
Evidence of increased lytic activity in SLCLs from AMS patients. (**A**) EA-D and Zta expression in AMS and SMS by flow cytometry (**B**) quantitation of EAD and Zta expression by flow cytometry (*p<0.05; one-way ANOVA followed by Tukey’s multiple comparison test) (**C**) Western blot of EBV latent (EBNA1, EBNA2, LMP1, EBNA3C) and lytic (Zta and EA-D) genes relative to β-actin. (**D**) Expression of EBV lytic (LC3, Zta) and latent (EBNA1, LMP1) genes by RT-qPCR.

The increased EBV DNA copy number and decreased proliferation and viability, and decreased expression of CD21 in SLCLs from AMS patients were suggestive of active EBV replication in these cell lines. We therefore next examined EBV lytic gene expression (EA-D and Zta) by flow cytometry (Figs. 2A-B) and found elevated expression of both lytic genes in AMS SLCLs compared to HC and SMS SLCLs and LCLs generated exogenously with lab strain EBV (B95.8 and Mutu). As expected, BJAB cells did not express either EA-D or Zta (Fig. 2B). Next, we confirmed these findings by Western blot, which demonstrated that EA-D and Zta could be detected in AMS SLCLs, but not in HC and SMS SLCLs, LCLs (B95.8) or BJABs (Fig. 2C). In addition to increased expression of Zta and EA-D, AMS4 demonstrated markedly decreased expression of the EBV latency membrane protein 1 (LMP1), Epstein-Barr nucellar antigen 3C (EBNA3C), and EBNA2 (Fig. 1C). This contrasts with the expected EBV gene expression pattern of LCLs (EBV latency III), which typically express all latency genes, including EBNA1, EBNA2, EBNA3C, and LMP1. RT-qPCR analysis of EBV genes also revealed that AMS cell lines have elevated expression of lytic genes, such as LF3 and Zta, compared to SLCLs from SMS and HC SLCLs (Fig. 2D). During the lytic cycle, LF3 RNAs are among the most abundant EBV transcripts expressed ^38^. LMP1 RNA was also elevated in AMS SLCLs.

### Whole genome sequencing of endogenous EBV from spontaneous LCLs

**Figure 3:**
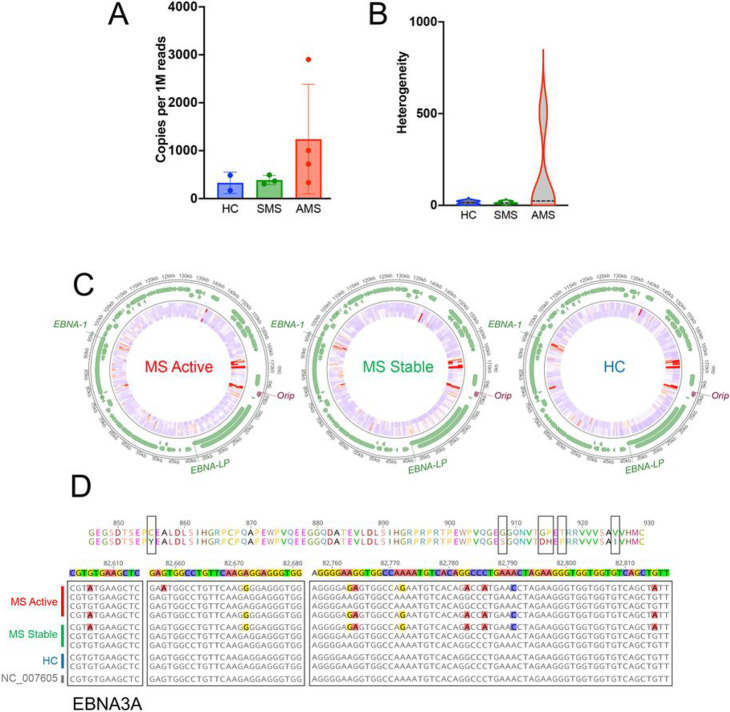
Whole genome NGS sequencing of endogenous EBV of SLCLs from AMS, SMS, and HC. (**A**) Copies of EBV genome per 1 M reads. (**B**) heterogeneity of EBV sequences found within samples from AMS, SMS, and HC. (**C**) AMS, SMS, and HC endogenous EBV aligned to the wild-type EBV genome (NC_007605.1). **(D)** Protein coding variations in EBNA3A identified in MS patients.

**Figure 4:**
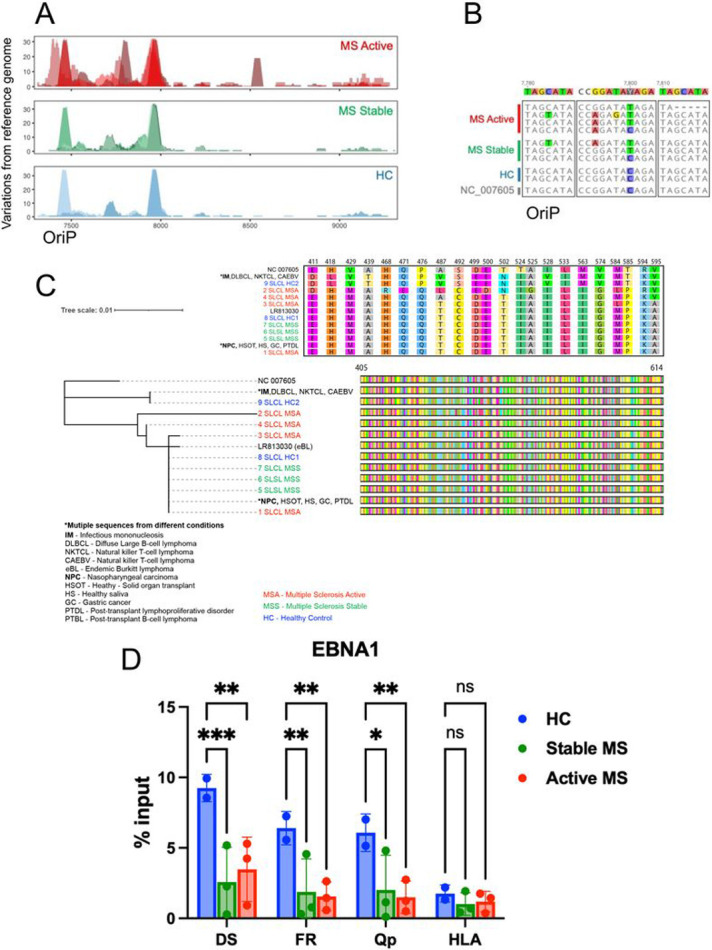
Heterogeneity in *oriP* and impaired EBNA1 binding in SLCLs from MS patients. (**A**) Heterogeneity in *oriP* visualized by the number of mutations compared to the reference. (**B**) Sequence alignment comparing variations in region 7780-7816 in *oriP*.(**C**) Phylogenetic analysis of *OriP*form SLCLs and other EBV associated diseases, including: infectious mononucleosis (IM), diffuse large B-cell lymphoma (DLBL), NK/T lymphoma, chronic active EBV (CAEBV), eBL (endemic Burkitt’s lymphoma), nasopharyngeal carcinoma (NPC), gastric cancer (EBVaGC), post-transplant lymphoproliferative disorder (PTDL), post-transplant B-lymphoma (PTBL). The *oriP* protein sequences for each category are shown in the upper panel. (**D**) ChIP assay for EBNA1 binding to the DS, Qp, and cellular locus CLIC1 in SLCLs. P values were determined for three biological replicates (***P < 0.001, ** P<0.01, *P<0.05; Two-way ANOVA). Immunoprecipitation was performed with IgG as a control (not shown).

Because of the observed differences in virus gene expression, we performed whole genome sequencing (Fig. 3C) to determine whether there were genomic differences in the endogenous EBV of SLCLs generated from AMS, SMS, and HC. In agreement with the viral load data, DNA-seq read counts revealed an increased copy number of EBV relative to cellular reads in AMS SLCLs compared to SLCLs generated from SMS or HCs (Fig. 3A). In addition, the degree of heterogeneity was increased in AMS (Fig. 3B), which may reflect a higher amount of EBV replication in these samples ^39^. Regions of increased heterogeneity include EBNA3 (Fig. 3C-D) and the *oriP* (Fig. 4A-C). Several polymorphisms of EBNA 3A were observed in three of the seven MS patients (Fig. 3C). The *oriP* region appears to be an additional region of heterogeneity, especially for MS patients (Fig. 4A-C). The *oriP* is a 1.7-kb region of EBV that is critical for the replication and stable maintenance of the virus in host cells. The *oriP* contains two essential regions, known as the dyad symmetry element (DS) and the family of repeats (FR) that contain multiple binding sites for EBNA1. DNA-seq revealed that EBNA1 binding sites within the FR element (genome coordinates 7780–7916) diverged from the reference sequence in 7/8 MS patient-derived genomes, while the HC sequences did not (Fig. 4B). We also found that EBNA1 protein sequence from AMS patients were not identical (Fig. 4C **top panel**), but they cluster with each other and the SMS samples, along with two reference samples including LR813030, which is likely from a Burkitt’s lymphoma and one HC sample (Fig. 4C **lower panel**). Additional reference samples and one HC form outgroups from virus isolated in other EBV-associated diseases (e.g. diffuse large B cell lymphoma, DLBCL; NK/T lymphoma; chronic active EBV, CAEBV; and nasopharyngeal carcinoma, NPC) (Fig. 4C). A recent report using next-generation sequencing to characterize EBNA1 sequence variation in an Australian cohort of MS patients also found low levels of variation in EBNA1 and suggested that although it is unlikely that there is an encephalitogenic strain of EBV, EBNA1 sequence variation does map to HLA-DRB1 binding sites, potentially effecting inflammatory and autoreactive T cell responses^40^.

To determine whether there are functional consequences of the EBNA1 sequence differences found in endogenous virus from MS patients, we performed a chromatin immunoprecipitation (ChIP) assay in SLCLs (Fig. 4D). EBNA1 binding to two EBV binding sites, including the DS and Qp and a known EBNA1 cellular binding site at the HLA locus, revealed decreased EBNA1 binding in MS patient SLCLs compared to HC SLCLs.

Several attempts have been made to associate EBV strains with clinical outcomes and geographic location ^40-42^. However, no specific EBV strains have been definitively associated with MS. Phylogenetic comparison of the genomes presented here to all available whole genome EBV sequences suggests that EBV sequences from these seven North American MS patients do not cluster together or distinctly apart from EBV sequenced from healthy controls. Therefore, this analysis from our cohort did not identify an MS-specific EBV strain (Supplemental Fig. 2A and B).

### Increased inflammatory gene expression in SLCLs generated from MS patients during active disease

**Figure 5:**
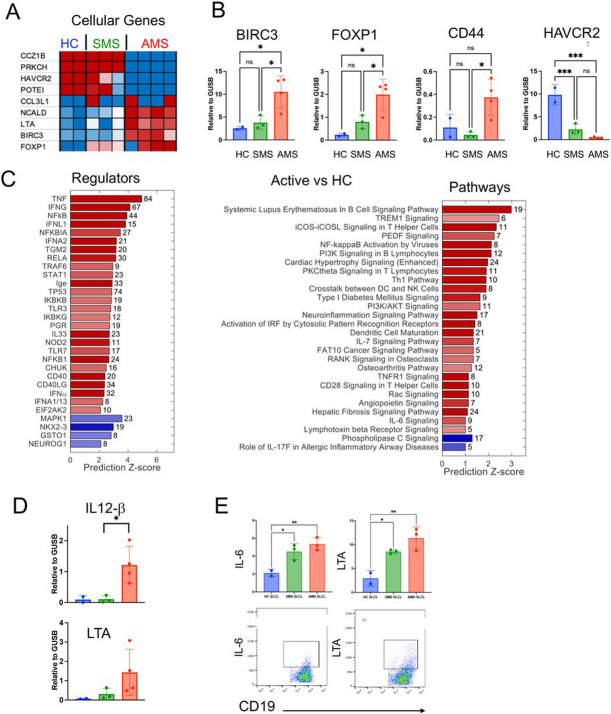
Increased inflammation in SLCLs generated from AMS patients. (**A**) RNA-seq showing top cellular genes that are upregulated (red) or downregulated in AMS, SMS, and HC SLCLs. (**B**) RT-qPCR for BIRC3, FOXP1, CD44, and HAVCR2. (**C**) Ingenuity pathway analysis showing top regulators of transcription and pathways that are upregulated or downregulated in AMS SLCLs compared to HC SLCLs. (**D**) RT-qPCR of IL-12β and LTA **E**) Intracellular cytokine staining for IL-6, GMCSF, LTA, and IL-10 in AMS, SMS, and HC SLCLS. (*p<0.05, **p<0.01, ***p<0.001; one-way ANOVA followed by Tukey’s multiple comparison test).

Increases in inflammatory responses are a hallmark of MS. Therefore, it was of interest to determine whether there were any differences in inflammatory proteins and gene pathways in SLCLs from MS patients compared to controls. First, we compared host gene expression profiles in SLCLs by RNA-seq and identified several cellular genes that were differentially expressed in SLCLs generated from MS patients (Fig. 5A). AMS cells had elevated expression of Baculoviral IAP repeat-containing protein 3 (BIRC3), involved in regulation of apoptosis, FOXP1, implicated in cellular differentiation, and CD44, implicated in cell adhesion and mobility. AMS cells showed decreased expression of Hepatitis A virus Cellular Receptor 2/TIM-3, which regulates T_reg_17 cells and may play a role in preventing autoimmunity (Fig. 5A). These RNA-seq results were confirmed by RT-qPCR (Fig. 5B). Ingenuity Pathway Analysis (IPA) revealed that regulators of transcription of several inflammatory proteins were upregulated in AMS SLCLs compared to HC SLCLs, including those associated with inflammatory cytokines (TNF, IFN-g, IFNa, IL-33), NFkB, and STAT1, while MAPK1, NKX2, GST01, and NEUROG1 were downregulated in AMS SLCLs (Fig. 5C). Regulators of STAT1 IFNL1, and IFNA2 were also upregulated in AMS compared to SMS SLCLs (Supplemental Fig. 3). Regulators of TNF and several other genes were increased in all MS patient SLCLs compared to HC SLCLs (Supplemental Fig. 4). Of interest, pathways associated with systemic lupus erythematosus (SLE), Th1 responses, IL-6 signaling, and neuroinflammation were among those pathways increased in AMS SLCLs compared to HC SLCLs (Fig. 5C). In addition, the coronavirus pathogenesis pathway, interferon signaling, IL-15 production and SLE pathway activation was increased in AMS SLCLs compared to SMS SLCLs (Supplemental Fig. 3), and NFkB pathways activated by viruses were increased in all MS SLCLs compared to HC SLCLs (Supplemental Fig. 3)

To confirm our RNA-seq findings and ingenuity pathway analysis suggesting SLCLs from MS patients have increased inflammatory phenotypes, we measured inflammatory cytokine responses by RT-qPCR and intracellular cytokine staining. IL12-b (a critical immunoregulatory cytokine that mediates Th1 commitment), LTA, and TNF-a were increased in AMS SLCLs by RT-qPCR (Fig. 5D), while IL-6 and LTA were increased in both AMS and SMS SLCLs compared to HC SLCLs by intracellular cytokine staining (Fig. 5E). Interestingly, the increase in LTA in AMS SLCLs was observed across all three assays (RNA-seq, RT-qPCR, and intracellular cytokine staining).

### Ganciclovir induces cell death during EBV lytic replication

**Figure 6:**
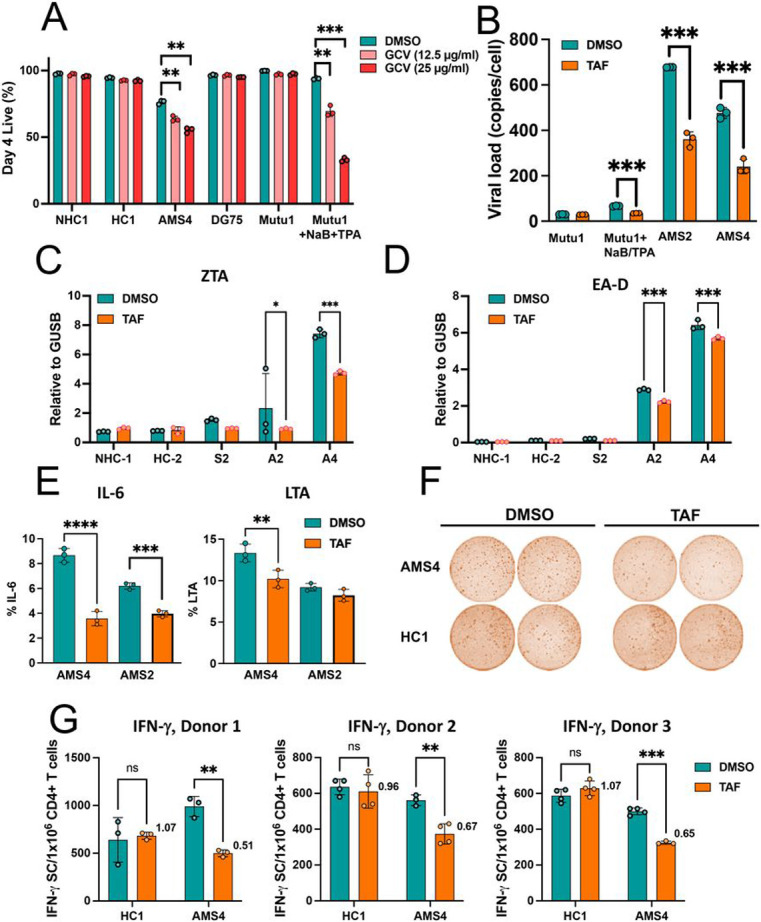
Tenofovir alafenamide (TAF) decreases EBV lytic activity and inflammation in SLCLs from MS patients. TAF (5μM) was added to SLCLs and LCLs (B95.8) for 72 hours with medium changed daily. (A) Cell death (% live) in cells treated with 12.5 or 25 μg/ml GCV with medium changed twice per day for four days. (B) EBV viral load in LCLs and SLCLs treated with TAF. (**D**) IL-6 and LTA expression by intracellular cytokine staining. (**C**) Zta expression by RT-qPCR. (**D**) EA-D expression by RT-qPCR. (**F-G**) Cytokine production during a mixed lymphocyte reaction as measured by EliSPOT. (**p<0.01, ***p<0.001; one-way ANOVA followed by Tukey’s multiple comparison test)

To determine the effect of antivirals known to target lytic replication, we treated a B95.8 transformed LCL (type III latency, NHC1), Mutu1 cells (type I latency), SLCLs (HC1 and AMS4), and a EBV- B cell line (DG75) with ganciclovir (GCV) and measured cell killing by the Zombie IR live/dead exclusion assay (Biolegend, San Diego, CA) after four days of treatment (Fig. 6A). Cell lines that demonstrate a type I (Mutu1) or type III (HC2, NHC1) latency pattern and DG75 cells were not susceptible to GCV mediated cell death. However, Mutu1 cells stimulated with TPA and sodium butyrate to induce active EBV replication became highly susceptible to GCV-mediated cell death. Interestingly, the SLCL line from patient AMS4 was also susceptible to GCV-mediated cell death in a dose dependent manner, although the SLCL from HC1 was not. These data suggest that there is ongoing expression of viral kinases and lytic cycle replication in the AMS4 cell line (Fig. 6A).

### Inhibition of EBV lytic replication reduces inflammatory phenotype

Recently, the nucleoside analogue tenofovir alafenamide (TAF) was demonstrated to inhibit the EBV DNA polymerase and inhibit EBV lytic replication ^32^. Therefore, it was of interest to determine whether TAF can inhibit EBV lytic activation in MS SLCLs and, consequently, decrease the inflammatory phenotype observed in SLCLs from patients with AMS. EBV viral loads were significantly decreased in MutuI cells that were TPA/NaB induced to enter the lytic cycle and SLCL lines AMS2 and AMS4 when they were treated with TAF (Fig. 6B), In addition, TAF significantly reduced lytic gene expression (Zta and EA-D) in SLCLs from AMS2 and AMS4. EA-D was also reduced in SMS2 (Fig. 6C-D). No difference in Zta or EA-D expression was observed when SCLSs from healthy controls or LCLs were treated with TAF (Fig. 6A-B). These data suggest that the effects of TAF on EBV copy number are specific to the EBV lytic cycle.

Because TAF reduced both EBV lytic gene expression and EBV viral loads, we were interested to determine if the TAF-mediated reduction in lytic activity and EBV lytic replication also reduced the levels of inflammation in these cells. Intracellular cytokine staining of two AMS SLCLs treated with TAF demonstrated a significant reduction in IL-6 expression in both lines and a significant decrease in intracellular LTA expression in AMS4 (Fig. 6E). We next performed a mixed T cell ELISpot assay and demonstrated that IFN-g T cell responses were decreased when T cells from three separate donors were cultured with SLCLs obtained from an MS patient in the active phase of their disease (Fig. 6E). These findings suggest that inhibition of a deregulated EBV lytic cycle reduces a B cell inflammatory phenotype in SLCLs from AMS patients. Moreover, concomitant with the decrease in EBV lytic activity, T cell responses to SLCLs from AMS patients were also decreased.

## Discussion

There are many well described abnormalities in both the humoral and cellular immune responses to EBV in patients with MS. The increased frequency of spontaneous immortalization of EBV infected B cells in MS patients compared to controls is an intriguing finding that was first described in the 1970s. However, intrinsic differences in virus gene expression and B cell inflammatory cytokine expression between the SLCLs from MS patients and controls have not been studied extensively. Here, we have used a rare collection of spontaneous lymphoblastoid cells (SLCLs) to study EBV gene expression patterns and to characterize host-virus interactions in the context of an individual’s endogenous EBV infection.

In this study, we demonstrate that SLCLs derived from patients during active disease had higher viral loads and increased lytic gene expression compared to SLCLs from healthy controls or MS patients with stable disease. This was shown by flow cytometry, RNA-seq, RT-qPCR and Western blot analysis of EBV lytic cycle genes. In related work (Monaco-Kushner-in submission), we reported that the frequency of EBV detection in CD19 + cells in AMS was twice that of the HC and SMS groups, providing further evidence for increased EBV replication during clinical relapse in MS. We also found that SLCLs from MS patients had increased production of inflammatory cytokines relative to healthy controls as measured by flow cytometry, RT-qPCR and RNAseq. Furthermore, treatment with TAF, an antiviral the reduces EBV lytic cycle gene expression reduced EBV lytic cycle gene activity in AMS cells, along with a reduction in B cell inflammatory gene expression and T-cell activation. Collectively, these data suggest that poor control of EBV latency in MS, particularly in the active phase of the disease, results in increased lytic gene expression and increased inflammation.

EBV immortalized B cells typically establish a stable type III latency gene program. This is thought to reflect the natural infection of EBV in vivo and the establishment of life-long latency in memory B cells. However, there is considerable variation of EBV gene expression at different stages of B-cell development and among EBV tumors, including varying frequency of sporadic lytic reactivation and “leaky” expression of lytic genes has been shown to occur ^39^. It is currently unclear if a specific EBV latency program contributes to the pathogenesis of MS and, therefore, it is of great interest to characterize the gene expression programs of SLCLs. Exogenously transformed LCLs, typically transformed with B95.8, maintain type III latency and stably express all latency genes including the EBNAs and LMP1, while lytic gene expression is negligible (Fig. 2C). We have demonstrated increased lytic EBV gene expression from MS patient SLCLs (Figs. 2A-C), particularly those obtained from PBMC acquired during “active” disease, indicating that these cells do not maintain type III latency and tend toward aberrant lytic activation. Moreover, the increased genomic heterogeneity observed is consistent with Increased lytic activation and virus replication in MS SLCLs cells (Figs. 3 and 4). ChIP analysis of EBNA1 binding at known viral and cellular regions indicates reduced EBNA1 binding in SLCLs derived from MS patients (Fig. 4D). We suggest that compromised EBNA1 binding contributes to the deregulation of type III latency, and a consequent increase in lytic gene expression and B-cell inflammatory response.

While increased inflammatory responses are well described in B cells from MS patients, it is unclear if EBV further exacerbates or drives these responses in the infected memory B cell population. Here, we have shown increased inflammatory cytokine expression and increased transcription of genes and transcriptional pathways involved in B cell inflammation, migration, maturation, and survival in AMS and SMS SLCLs compared to HC SLCLs. While these findings are generally expected in MS samples compared to controls, the role of EBV in skewing MS patient B cells toward a pathway of inflammation is incompletely characterized. Tenofovir alafenamide (TAF), a nucleoside analogue reverse-transcriptase inhibitor specifically designed to inhibit the replication of the human immunodeficiency virus and hepatitis B virus, was recently shown to have activity against EBV ^32^. In addition, MS patients are reported to benefit from anti-retroviral therapy that includes TAF ^43^. We were, therefore, interested to determine if TAF would inhibit EBV lytic activation and if we would observe a concomitant decrease in inflammatory cytokine production. Our studies indicate that TAF does indeed decrease lytic activity as measured by decreased expression of EBV lytic genes (Zta and EA-D, Fig. 6A-B) and decreased viral loads (Fig. 6C). In addition to the observed decrease in inflammatory cytokines produced by AMS SLCLs treated with TAF, we also found reduced induction of NF-a and IFN-g in T cells exposed to SLCLs in our mixed T cell ELISpot assay (Fig. 6E). These data indicate that the abnormal EBV lytic activation in AMS SLCLs is capable of driving inflammation in both memory B cells and T cells, suggesting that EBV infected B cells may be a source of pathologic immune responses in both B and T cells and that this inflammatory response can be suppressed by inhibiting lytic activation.

The disease-modifying effects of B cell depletion therapies have underscored the importance of understanding the role of inflammatory B cells in the pathogenesis of MS, but do not specifically elucidate the role of EBV in driving disease pathogenesis. Our findings suggest that poorly controlled EBV latency and EBV lytic activation has the potential to drive inflammatory, pathogenic B cand T cell responses that can contribute to the inflammatory milieu and pathobiology of MS. In addition, this study provides support for developing therapies that specifically target EBV infected memory B cells in MS. In addition to TAF, which effectively inhibits EBV lytic infection, several other treatment modalities for targeting EBV infected B cells are in development, including autologous T-cell therapies to compensate for deficient CTL control of EBV infected B cells ^44-47^. By specifically targeting EBV infection, these agents have the potential to achieve clinical efficacy in MS while further illuminating EBV-driven mechanisms of neuroinflammation and disease pathogenesis.

There are several limitations to our study, including the use of non-HLA matched T-cells in mixed reactions with SLCLs. However, these studies focused on the effects of TAF for each donor T-cell reactions to the same SLCLs. Moreover, our cohort of SLCLs remains small. While there are ongoing efforts to increase this size, the success rate of obtaining SLCLs from age matched healthy donors is extremely low and may suggest that it is rarer to generate SLCLs from young, healthy individuals and becomes easier as immune control wanes during normal aging. Nevertheless, our study of these few SLCLs has provided plausible mechanistic basis for EBV as not only as trigger, but also a driver of B cell pathogenesis in MS.

## Materials And Methods

### Patient information

Fourteen healthy controls (HC), seven MS patients with stable disease (SMS), and eight MS patients with active disease (AMS) were enrolled in this study. AMS was defined as the presence of at least one cerebral enhancing lesion (CEL) at the time of blood collection; patients with stable disease (SMS) had no CEL at the time of sampling (Table 1). Blood from HC was collected from volunteers at the Transfusion Medicine Blood Bank of the National Institutes of Health (Bethesda, MD). All MS patients were enrolled in an IRB-approved natural history study. Both HC and MS patients signed informed consent prior to participation. Donors for the mixed lymphocyte reaction experiments were obtained from The Wistar Institute’s blood donation program.

### Cell Isolation Generation of Spontaneous Lymphoblastoid Cell lines (SLCLs) and tissue culture conditions

Peripheral blood mononuclear cells (PBMCs) from HC donors and MS patients were isolated from whole blood by ficoll-hypaque density centrifugation and cryopreserved in freezing media containing 20% DMSO. SLCLs were generated by spontaneous expansion from PBMCs in the presence of cyclosporin A to eliminate T cells ^48^. Briefly, PBMCs were thawed in growth medium [RPMI-1640 medium (Gibco, Gaithersburg, MD) with 10% fetal bovine serum (FBS; Gibco, Gaithersburg, MD), 1% gentamicin (50mg/ml; Quality Biological, Gaithersburg, MD), and 1% L-Glutamine (200mM; Quality Biological, Gaithersburg, MD)] and then plated in 96-well round bottom plates at a density of 1x10^6^/well in a total volume of 200μl. Once per week, 100μl medium was removed and replaced with fresh growth medium. After 3 weeks, a final concentration of 2μg/mL of cyclosporin A (Sigma-Aldrich, St. Louis, MO) was added to each well (Fig. 1A). Three to six weeks later, clusters of B-lymphoblastoid cells (i.e., spontaneous lymphoblastoid cell lines; SLCLs) emerged (Fig. 1A) and were transitioned into progressively larger tissue culture wells until they were robust and numerous enough to be cultured in T_25_ flasks. Once established, SLCLs were maintained in growth medium and incubated at 37°C under a 5% CO_2_ humidified atmosphere.

### Carboxyfluoresein succinimidyl ester (CFSE) Proliferation Assay

SLCLs were labeled with 1.5 μM CFSE (Invitrogen, Waltham, MA) and incubated for seven days. CFSE dilution was measured by flow cytometry using a BD-LSR II (BD Biosciences; Bedford, MA) and proliferation indices were calculated using FlowJo software (Ashland, OR).

### Cell viability assay

SLCLs, LCLs (B95.8 transformed), and an EBV (−) cell line (BJABS) were plated in growth medium at 8 x 10^3^ cells (in 100 μl) per well in an opaque 96-well plate. The Cell Titer-Glo^®^ Cell luminescent Cell Viability Assay (Promega, Madison, WI) was used as per kit instructions. Briefly, Cell Titer-Glo^®^ substrate and buffer were combined and 100 μl of this mixture (1:1 ratio of Cell Titer-Glo^®^: cell mixture) was added to each well and mixed. The plates were then incubated for 20 minutes (for cell lysis to occur), and luminescence was measured on a CLARIOstar^plus^ (BMG Labtech, Ortenberg, Germany) multilabel plate reader to determine cell viability; the generation of a luminescent signal is proportional to the amount of ATP present. This assay was performed on Day 2 and Day 4 after plating and the percent increase in luminescence in 48 hours (between Days 2 and 4) was calculated.

### EBV viral load

DNA was isolated from SLCLs, LCLs (B95.8 transformed LCLs; one from an MS patient, one from a HC), and BJABS (EBV negative control cell line) using a DNeasy blood and tissue kit (Qiagen, Hilden, Germany). PCR was used to quantify EBV viral load using a standard curve generated with Namalwa cells (two copies of EBV per cell). Primers used include: EBNA1 F 5’- TCA TCA TCA TCC GGG TCT CC-3’; EBNA1 R 5’- CCT ACA GGG TGG AAA AAT GGC – 3’; b-actin F 5’- GCC ATG GTT GTG CCA TTA CA-3’; b-actin R 5’- GGC CAG GTT CTC TTT TTA TTT CTG-3’.

### Extracellular staining for cell surface markers and EBV lytic genes

For extracellular staining, cells were treated with a human FcX blocker antibody mix (BioLegend, San Diego, CA) for 20 min. Cells were then washed with FACS buffer (PBS, 5% FBS, 0.1% NaN3 sodium azide) and centrifuged at 1,500 rpm for 5 minutes before staining for 30 minutes at 4°C with the appropriate cocktail containing antibodies to one or more of the following: CD19, CD20, CD21, CD11c, CD80, CD86, EA-D, and/or ZTA. After incubation with antibodies for cell surface markers, cells were washed with FACS buffer, centrifuged at 1,500 rpm for 5 minutes, and then resuspended in 500 μl FACS buffer for immediate analysis by flow cytometry using a BD-LSR II (BD Biosciences, Bedford, MA). Compensation was determined using CompBeads (BD Biosciences, Bedford, MA) and single stained fluorescent samples. Doublet discrimination (FSC-H: FSC-A) was performed, and dead cells were excluded using the live/dead discrimination dye Zombie IR (BioLegend, San Diego, CA) before analysis using FlowJo software (Ashland, OR).

### Western blot for EBV latent and lytic genes

Cell lysates were prepared in radioimmunoprecipitation assay (RIPA) lysis buffer (50 mM Tris-HCl, pH 7.4, 150 mM NaCl, 0.5% sodium deoxycholate, 0.1% SDS,1 IGEPAL, 10mM glycero-phosphate, 1mM sodium phosphate, 1mM sodium orthovanadate, 1 mM EDTA) supplemented with phenylmethysolfonyl fluoride, benzonase, and 1× protease inhibitor cocktail (Thermo Scientific, Waltham, MA). Protein extracts were obtained by centrifugation at 3,000 × *g* for 10 min at 4°C. Protein concentrations were equalized using a bicinchoninic acid (BCA) protein assay (Pierce, Appleton, WI) and lysates were subsequently boiled with 2× Laemmli sample buffer (Bio-Rad, Berkley, CA) with 2.5% β-mercaptoethanol (Sigma-Aldrich, St. Louis, MO). Proteins were resolved by gel electrophoresis on an 8–16% Tris-glycine precast gel (Invitrogen, Waltham, MA) and transferred to an Immobilon-P membrane (Millipore, Burlington, MA). Membranes were blocked in 5% milk in PBS-T for 1 h at room temperature and incubated overnight at 4°C with primary antibodies against EBNA1 (EBS-I-024; Thermo Scientific, Waltham, MA), EBNA2 (MABE8; Millipore, Burlington, MA), LMP1 (M0897; Dako, Santa Clara, CA), EBNA3C (LS-C14045; LS Bio, Seattle, WA), EAD (ab30541; Abcam, Cambridge, United Kingdom), and b-actin (A3854; Sigma-Aldrich, St. Louis, MO) as recommended per the manufacturer. Anti-Zta antibody was generated in-house. Membranes were washed, incubated for 1 h with the appropriate secondary antibody, either goat anti-rabbit IgG-HRP rabbit anti-mouse IgG-HRP or anti-sheep IgG. Membranes were then washed and detected by enhanced chemiluminescence.

### EBV and host gene expression analysis

RNA was isolated from SLCLs using the RNeasy Mini Kit (Qiagen, Hilden, Germany) and treated with deoxyribonuclease (Qiagen, Hilden, Germany). Reverse transcription followed by real-time quantitative PCR (RT-qPCR) was used to measure EBV gene expression levels. Primers used for qPCR of EBV genes include: EBNA1 F (5’-GGTCGTGGACGTGGAGAAAA-3’), EBNA1 R (5’-GGTGGAGAC CCGGATGATG-3’); Zta F (5-TCTGAACTAGAAATAAAGCGATACAAGAA-3’), Zta R (TTGGGCACATCTGCTTCAAC); EA-D F (5’-TTGGGCAGGTGCTGTTGAT-3’), EA-D R (5’- TGCCCACTTCTGCAACGA-3’); LMP1 F (5’-TCCAGAATTGACGGAAGAGGTT-3’), LMP1 R (5’-GCCACCGTCTGTCATCGAA-3’); LF3 F(5’-GCCAATAACTACCTGCCCCT); LF3 R(5’-AGACTTTCGGGGCATTGGTG-3’). Primers used for qPCR of host genes include: IL12B F (5’-TGCCCTGCAGTTAGGTTCTG), IL-12B R(5’-TGGGTCTATTCCGTTGTGTCT-3’), FOXP1 F(5’-GTGGCAAGACAGCTCCTTCT-3’), FOXP1 R (5’-ATAGCCACTGACACGGGAAC-3’; HAVCR2 F(5’-GCATCTACATCGGAGCAGGG-3’), HAVCR2 R(5’-TTGGCCAAAGAGATGAGGCTTA-3’); BIRC3 F(5’-GACTGGGCTTGTCCTTGCT-3’), BIRC R (5’-GAAGAAGTCGTTTTCCTCCTTTG-3’). Glucuronidase beta (GUSB) was used as a cellular control: GUSB (5’-CGCCCTGCCTATCTGTATTC and 5’-TCCCCACAGGGAGTGTGTAG-3’). The average cycle threshold (CT) was determined by three independent samples. Template-negative (quantitative PCR reaction mixtures without cDNA) and RT-negative (RNA after genomic DNA elimination) conditions were used as controls. All data were normalized to the housekeeping gene *GUSB*.

### Whole genome sequencing and analysis

DNA was extracted from SLCLs using the DNeasy Blood and Tissue Kit (Qiagen, Hilden, Germany). The Illumina DNA preparation kit was used for sequencing library preparation according to the manufacturer’s instructions. Sequencing was done with an Illumina NextSeq 500 system in high-output mode to generate a total of ~4 × 10^8^ paired-end 75-bp reads. Fastq reads were aligned against the EBV genome (accession NC_007605.1) using the BWA algorithm ^49^. Analysis of heterogeneous sites was performed following the procedure described by Čejková et al. ^50^. The consensus EBV genome for each sample was extracted using samtools^51^. Circus plots were generated using the R BioCircos package. Proteins were translated from nucleotide sequences using Geneious prime 2022.2 (https://www.geneious.com). Nucleotide and protein sequences were aligned using MAFFT ^52^v7.407 and visualized using Geneious prime 2022.2. Reference EBNA1 regions, in addition to the sequence from NC_007605.1, were obtained by querying each SLCL-derived sequence against nr using tblastn and taking differentially occurring sequences between matches for the three groups of samples (HC, AMS, SMS). One representative for each of three groups of identical sequences was kept. A phylogenetic tree of the 13 resulting sequences (9 samples, 4 reference) was constructed using PhyML with default parameters and visualized using iTOL^53,54^.

### Phylogenetic Analysis

A Maximum Likelihood tree (Supplemental Fig. 2A) was built using all available complete genomes and the study consensus sequences. After performing a quality control check, genomes were aligned by MAFFT using NC 007605 as a reference. A maximum likelihood phylogeny was inferred using RAxML v8.2.4 using a GTR substitution model^55,56^accounting for among-site rate heterogeneity using the Γ distribution and four rate categories (GTRGAMMA model)^57^ for 100 individual searches with maximum parsimony random-addition starting trees. Node support was evaluated with 100 nonparametric bootstrap pseudoreplicates^58^. The initial ML newick tree and the whole-genome alignment, were used as input for ClonalframeML to infer recombination using 100 pseudo-bootstrap replicates^59,42^ For better visualization, the tree was edited using iTol website (v4.2.3) and Microreact (v214)^60,61^.

### Chromatin immunoprecipitation (ChIP) assay

We previously described a chromatin immunoprecipitation (ChIP) assay to measure EBNA1 protein binding to viral and cellular DNA elements in AMS, SMS, and HC SLCLs ^62^. Briefly, SLCLs were harvested and cells were crosslinked in 1% formaldehyde for 15 min, followed by quenching for 5 min with 0.125 M glycine, and then lysed in 1 ml SDS lysis buffer (1% SDS, 10 mM EDTA, and 50 mM Tris-HCl, pH 8.0) containing 1 mM PMSF and protease inhibitor cocktails (Sigma-Aldrich, St. Louis, MO), and kept on ice for 10 min. Lysates were sonicated with a Diagenode Bioruptor, cleared by centrifugation to remove insoluble materials, diluted 10-fold into IP Buffer (0.01% SDS, 1.1% Triton X-100, 1.2mM EDTA, 16.7mM Tris pH 8.0, 167mM NaCl, 1 mM PMSF, and protease inhibitors cocktail), and incubated with rabbit anti-EBNA1 antibody (2 μg/reaction) or IgG control for immunoprecipitation overnight at 4°C before washing five times in wash buffer at 4°C and eluting with 150 μl Elution buffer (10mM Tris, pH 8.0, 5mM EDTA, and 1% SDS) at 65°C for 30 min. The elutes were then incubated at 65°C overnight to reverse cross-linking, and further treated with Proteinase K in a final concentration of 100 μg/ml at 50°C for 2 hrs. ChIP DNA was purified by Quick PCR Purification Kit (Life Technologies, Waltham, MA) following the manufacturer’s instruction. Real-time quantitative PCR (ABI 7900HT Fast Real-Time PCR System; Applied Biosystems, Waltham, MA) was performed on ChIP DNA to quantitate two EBV loci (DS50 and Qp) and one cellular locus (CLIC1), which have been reported to associate with EBNA1. Results were quantified as % input. Primer sets used for ChIP were as follows: DS50 F (5’- ATGTAAATAAAACCGTGACAGCTCAT-3’), DS50 R (5’- TTACCCAACGGGAAGCATATG-3); QP F (5’- AAATTGGGTGACCACTGAGGGAGT-3’), QP R (5’-ATAGCATGTATTACCCGCCATCCG-3’); CLIC1 F (5’-CCTAAGCTGAGGGTGATTCATCTC-3’), CLIC1 R (5’-CCCCACATCCTTGACAGGAA-3’).

### RNA Seq and RNA-Seq

Total RNA was isolated from 2 × 10^6^ cells using the RNeasy Mini Kit (Qiagen, Germany) and treated with deoxyribonuclease (Qiagen, Hilden, Germany), following the manufacturer’s protocol. RNA samples were submitted to the Wistar Institute genomics core facility for initial analysis of RNA quality, with each sample having an RNA integrity number (RIN) value greater than 8.5 (TapeStation; Agilent Technologies, Santa Clara, CA). Sequencing library preparation for SLCLs was completed using the ScriptSeq RNA-seq library preparation kit. Sequencing was performed with an Illumina NextSeq 500 system in high-output mode to generate ~140 × 10^6^ reads (2 × 75 bp) across three multiplexed and pooled samples. The QuantSeq 3’-mRNA kit (Lexogen) was used to generate Illumina-compatible sequencing libraries according to the manufacturer’s instructions. Sequencing was done with an Illumina NextSeq 500 system in high-output mode to generate ~4 × 10^8^ reads (1 × 75 bp) across 12 multiplexed and pooled samples.

RNA-seq data was aligned using the bowtie2 ^63^ algorithm against the hg19 human reference genome and RSEM v1.2.12 software ^64^ was used to estimate read counts and RPKM values using gene information from Ensemble transcriptome version GRCh37.p13^65^. DESeq2 was used on raw counts to estimate significance of expression differences between any two experimental groups and generate normalized counts. Gene set enrichment analysis was done using QIAGEN’s Ingenuity^®^ Pathway Analysis software (IPA^®^; QIAGEN, Redwood City, CA; www.qiagen.com/ingenuity) using “Canonical pathways” and “Upstream regulators” option. Top significant results with at least 4 genes and with predicted activation state Z-score of at least ∣Z∣>1 were reported.

### Intracellular Cytokine Staining

Phorbol myristate acetate (PMA; 20 ng/ml, Sigma-Aldrich), Ionomycin (500 ng/ml, Sigma-Aldrich), and Golgi stop (Monensin, BD Bioscience, East Rutherford, NJ) were added to 1x10^6^ cells four hours prior to staining with live/dead reagent to exclude dead cells (Zombie/NIR, Biolegend, San Diego, CA) and anti CD19(BD Biosciences, East Rutherford, NJ). Cells were subsequently fixed and permeabilized with fixation/permeabilization buffer (BD Bioscience, East Rutherford, NJ). Antibodies for IL-6 and LTA (BD Bioscience, East Rutherford, NJ) were added and incubated for 30 min on ice. Samples were then washed twice and analyzed by flow cytometry with a BD-LSR II (BD Biosciences, Bedford, MA).

### Mixed lymphocyte reactions

Whole blood was collected from an individual donor and immediately diluted 1:1 with sterile DPBS supplemented with 2% FBS. Next, 15ml of Lymphoprep (STEMCELL Technologies, Vancouver, CA) was added through the insert hole of SepMate-50 columns (STEMCELL Technologies, Vancouver, CA), and 30ml of diluted blood was slowly layered over top. Tubes were then spun at 1200 x g for 10 mins at room temperature with full brake (as per manufacturer’s instructions). Supernatant was then transferred to a normal 50ml conical tube, being careful to minimize transfer of the loosely packed erythrocyte layer. Supernatant volume was increased to 50ml with DPBS/2% FBS and spun at 500 x g for 10mins. The supernatant was then removed, and wash was repeated a minimum of 4 additional times. Prior to the final wash, total numbers of peripheral blood mononuclear cells were counted. CD4 T cells were isolated from PBMC using Dynabeads Untouched Human CD4 T cells kit following the manufacturer’s protocol (Invitrogen, Waltham, MA). CD4 T cells were diluted such that 25,000 cells were added per 100 μl in each well of a 96-well plate. HC1 and AMS4 LCLs were treated with 50 μg/ml mitomycin C (Sigma-Aldrich, St. Louis, MO) for 20 min at 37°C, washed three times, and diluted such that 5,000 cells were added per 100 μl in each well of a 96-well plate. The use of mitomycin C prevented HC1 and AMS4 LCLs from overgrowing the culture. CD4 T cells were cultured alone (Negative), with anti-CD3/CD28 Dynabeads Human T-Activator (Thermo Fisher Scientific, Waltham, MA) (Positive), or with 5,000 HC1 or AMS4 SLCLs in RPMI medium −/+ TAF for 6 days. Cell viability was determined using the CellTiterGlo assay (Promega, Madison, WI) at day 6.

### ELI Spot

HC1 and AMS4 LCLs were treated with 50 μg/ml mitomycin C as described above, and co-cultured with PBMC −/+ TAF for 7 days. Then CD4 T cells were isolated from each group using Dynabeads Untouched Human CD4 T cells kit following the manufacturer’s protocol (Invitrogen, Waltham, MA) and seeded at a density of 100,000 cells/well to 96-well ELISpot-plates (Millipore, Burlington, MA) pre-coated with either TNF-α ELISpot Development Module (SEL210, R&D Systems, Inc.) or IFN-γ ELISpot Development Module (SEL285, R&D Systems, Inc.). Cells were incubated at 37°C for 24h, after which cells were removed and proceeded to color development with ELISpot Blue Color Module following the manufacturer’s protocol (SEL002, R&D Systems, Inc., Minneapolis, MN). Color development was stopped with a water wash and the plate was air-dried overnight at RT away from light. Spots were enumerated using an automated spot counter (ImmunoSpot^®^ CTL S6 Micro Analyzer; Cellular Technology Limited, Shaker Heights, OH).

## Figures and Tables

**Table 1.** Patient Information

Sample	Treatment	Age	Sex/Race	Sex	MRI (CEL)	EDSS	Successful Generation of SLCL (%)
Healthy Controls							2/14 (33%) ^*^
HC1	N/A	67	M/WC	M	N/A	N/A	
HC2	N/A	60	M/A	M	N/A	N/A	
SMS							3/7 (43%)
SMS1	DMT	63	M/WC	M	0	7	
SMS2	Untreated	43	F/WC	F	0	1.5	
SMS3	Copaxone	67	M/WC	M	0	1	
AMS							4/8 (50%)
AMS1	Rebif (IFN-P1)	28	F/WC	F	2	1	
AMS2	DMT	43	M/WC	M	1	2	
AMS3	Terifluonomide	35	F/B/AA	F	1	1	
AMS4	DMT	46	F/B/AA	F	1	6	

CEL= cerebral enhancing lesion; HC=healthy control; AMS=MS (active); SMS (stable); N/A= not applicable; DMT= disease modifying therapy; M= male; F= female; EDSS= expanded disability status scale; WC=white/Caucasian; A=

Asian; B/ AA= black/African American

* P=0.0464, Fisher's Exact Test
